# Social Islands: Examining the Intersection of Isolation and Technology Use

**DOI:** 10.1093/geroni/igab046.1781

**Published:** 2021-12-17

**Authors:** Joseph Svec, Megan Gilligan

**Affiliations:** 1 Iowa State University, Ames, Iowa, United States; 2 Iowa State University, Iowa State University, Iowa, United States

## Abstract

Life course theory suggests that social relationships are tied to overall well-being. In the extant literature social isolation negatively impacts physical and mental health outcomes in later life. In this study, we focus specifically on the association between social isolation and older adult’s self-rated health status. We also examine whether and the extent to which technology use augments negative health consequences attributed to isolation. Using data on 3,758 older adults (ages 65+) from the 2018 wave of the Health and Retirement Study, we contribute to current scholarly examinations at the intersection of technology and isolation. We conduct a series of ordinal logistic regressions to estimate the odds of respondents’ higher self-rated health (from poor = 1 to excellent = 5) on subjective measures of social isolation (i.e. feel left out, lack of companionship and isolated from others) in addition to whether respondents live with a partner and have an adult child who lives in close geographic proximity. Preliminary results show that individuals who perceived higher levels of social isolation evaluated their own health status as poorer. We also find that the use of computers for virtual communications corresponds with higher self-rated health statuses, regardless of the proximity of children or other family members. However, a negative interaction between computer use and isolation indicates the positive effects of technology are limited for those who are highly isolated. These findings suggest that technology impacts on health are nuanced, where an overreliance on technology as a substitute may not consistently yield positive outcomes.

